# Visualization of Freezing Process *in situ* upon Cooling and Warming of Aqueous Solutions

**DOI:** 10.1038/srep07414

**Published:** 2014-12-10

**Authors:** Anatoli Bogdan, Mario J. Molina, Heikki Tenhu, Erminald Bertel, Natalia Bogdan, Thomas Loerting

**Affiliations:** 1 Institute of Physical Chemistry, University of Innsbruck, Innrain 80-82, A-6020, Innsbruck, Austria; 2 Laboratory of Polymer Chemistry, Department of Chemistry, University of Helsinki, P.O. Box 55, FIN-00014, Helsinki, Finland; 3 Department of Physical Sciences, University of Helsinki, P.O. Box 64, FI-00014, Helsinki, Finland; 4 Department of Chemistry and Biochemistry, University of California, San Diego, La Jolla, CA 92093-0356, USA; 5 Faculty of Medicine, University of Helsinki, P.O. Box 63, FIN-00014, Helsinki, Finland

## Abstract

The freezing of aqueous solutions and reciprocal distribution of ice and a freeze-concentrated solution (FCS) are poorly understood in spite of their importance in fields ranging from biotechnology and life sciences to geophysics and climate change. Using an optical cryo-miscroscope and differential scanning calorimetry, we demonstrate that upon cooling of citric acid and sucrose solutions a fast freezing process results in a continuous ice framework (IF) and two freeze-concentrated solution regions of different concentrations, FCS_1_ and FCS_2_. The FCS_1_ is maximally freeze-concentrated and interweaves with IF. The less concentrated FCS_2_ envelops the entire IF/FCS_1_. We find that upon further cooling, the FCS_1_ transforms to glass, whereas the slow freezing of FCS_2_ continues until it is terminated by a FCS_2_-glass transition. We observe the resumed slow freezing of FCS_2_ upon subsequent warming. The net thermal effect of the resumed freezing and a reverse glass-FCS_1_ transition produces the *T_tr2_*-transition which before has only been observed upon warming of frozen hydrocarbon solutions and which nature has remained misunderstood for decades.

Liquid water, arguably the most important solvent on Earth, rarely occurs in pure state but rather as a component of aqueous solutions. In contrast, the solid form of water, ice, is highly intolerant to impurities^1^. Hence, upon freezing aqueous solutions separate into pure ice and a FCS which vitrifies^2^,^3^,^4^,^5^,^6^,^7^,^8^,^9^,^10^,^11^,^12^ or freezes^13^,^14^ upon further cooling. This phase separation and FCS distribution within the ice play an important role in various natural, industrial and biotechnological processes. For example, FCS veins/pockets within the ice affect microbial activity in ice sheets^15^, hydromechanics of freezing soils^16^, rheology and transport properties of glaciers^17^,^18^ and sea ice^19^,^20^. In the atmosphere, FCS around cloud ice particles affects physical and chemical properties of cirrus clouds^21^,^22^ and the rate of stratospheric ozone destruction^22^,^23^ and, consequently, impacts the climate. When living matter freezes, growing extra- and intracellular ice disrupts cell membranes and this together with other freeze-induced stresses (the formation of FCS, cellular dehydration, etc.) is fatal to cells^24^,^25^. Freeze-induced separation is crucial in freeze-desalination of sea water^26^, freeze-purification of waste water^27^, food industry^8^,^9^,^10^,^11^,^28^,^29^,^30^,^31^ and biotechnology, particularly, in freeze-drying (lyophilization) which is used to extend the stability and shelf life of foods^8^,^9^,^10^,^11^,^28^,^29^,^30^,^31^ and labile drugs, especially pharmaceutical proteins^2^,^3^,^4^,^5^,^6^,^7^,^32^, because degradation reactions are decelerated in lyophilized products^2^,^3^,^4^,^5^,^6^,^7^,^8^,^9^,^10^,^11^,^28^,^29^,^30^,^31^,^32^.

Lyophilization is a time- and energy-intensive process which besides freezing consists of primary drying, sometimes preceded by annealing^33^, and secondary drying^2^,^3^,^4^,^5^,^6^,^7^,^33^,^34^,^35^,^36^,^37^ performed upon subsequent warming. The duration of drying is largely determined by the freezing step^33^,^34^,^35^,^36^,^37^. Vitrified or crystallized FCS creates a solid matrix suitable for drying. The morphology of the ice/FCS-matrix controls product resistance to vapour flow of sublimated ice during primary drying, desorption of residual water from a resulting porous cake during secondary drying, and the quality attributes of final lyophilized products such as product porous structure, physical state, residual moisture, reconstitution time, etc.^2^,^3^,^4^,^5^,^6^,^7^,^33^,^34^,^35^,^36^,^37^. Freezing methods impose constraints on ice/FCS-matrix morphology. Methods which involve small formulation supercoiling and small cooling rate, produce fewer and larger ice crystals which makes primary drying faster and leaves larger pores in a cake after ice sublimation^2^,^3^,^4^,^5^,^6^,^7^,^33^,^34^,^35^,^36^,^37^. However, the genuine ice/FCS morphology formed during freezing is not known. Currently, it is believed that freezing produces ice crystals embedded and dispersed in a matrix of glassy and/or crystallized FCS^2^,^3^,^33^,^34^,^35^,^36^,^37^. However, *such seeming picture* of ice/FCS morphology cannot account for the appearance of two transitions, *T_tr1_* and *T_tr2_*, calorimetrically observed upon warming of frozen carbohydrate solutions^9^,^10^,^11^,^12^,^29^,^30^,^31^,^38^,^39^. The cold transition, *T_tr1_*, is usually related to a glass transition of FCS. Hitherto the nature of the warm transition, *T_tr2_*, and the question of whether *T_tr1_* or *T_tr2_* should be related to the glass transition of *maximally* FCS, *T_g_*', has remained a subject of debate for decades^9^,^10^,^11^,^12^,^29^,^30^,^31^,^38^,^39^. The knowledge of *T_g_*' is important for the determination of collapse temperature, *T_c_*, at which lyophilized products start losing their amorphous structure^40^,^41^. The primary drying is performed at a product temperature, *T_p_*, which is slightly below *T_c_* ≈ *T_g_'* + 2 K^3^,^40^.

Visualization of the freezing process *in situ* would reveal the genuine morphology of ice/FCS. Unfortunately, using an optical cryo-microscope (OC-M), the freezing of bulk solutions is seen as an abrupt black flash because of light scattering from numerous rapidly formed ice crystals^42^. Finding methods for observing the freezing process *in situ* is challenging but would crucially improve our knowledge of the freezing phenomenon and understanding of the variety of natural and biotechnological processes. In this work, we observe the freezing process of ‘2-dimensional' samples (5–10 μm films) of citric acid (CA) and sucrose solutions *in situ* with an OC-M. The term “2-dimensional” is used in the following solely as a shorthand to discriminate the samples used in OC-M from those in DSC. It does not imply a different dimensionality of the physics, such as different nucleation behavior or different dimensionality of the growing ice-network, since a thickness of 5–10 μm is still large in comparison to the size of a critical nucleus and large in comparison to the characteristic dimension of the ice structures observed in OC-M. The choice of solutes was motivated by the fact that CA is widely used in food industry, pharmaceutics^43^,^44^, tissue engineering^45^, and sucrose, being a natural lyoprotectant, is important in life sciences^12^, food industry^9^,^10^,^11^,^29^,^30^,^31^, biotechnology^38^,^39^ etc. We also investigate ‘3-dimensional' (bulk) samples of the same solutions with differential scanning calorimetry (DSC). The obtained OC-M and DSC results are mutually complementary and give a clear picture of the freezing process and formed ice/FCS morphology.

## Results

**Figure 1:**
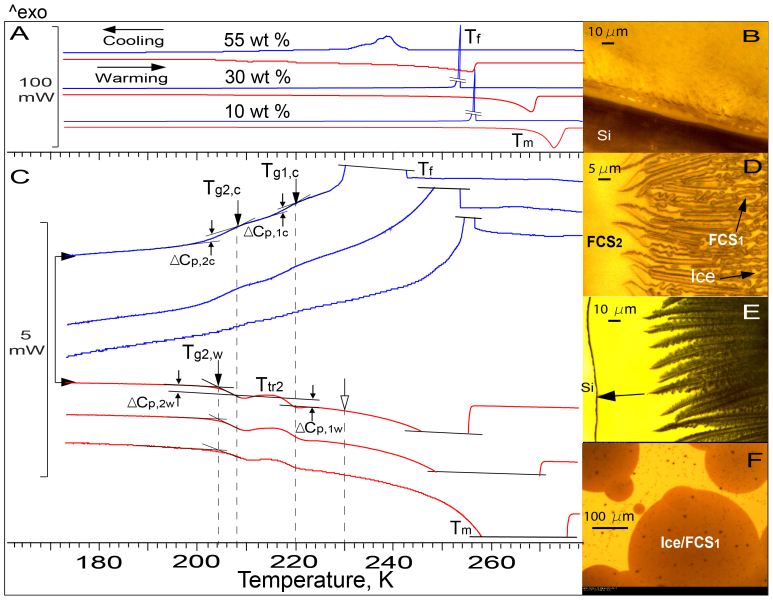
DSC thermograms and OC-M images of CA/H_2_O. (A). Upper blue and lower red lines are cooling and warming thermograms, respectively. Skewed lines truncate freezing peaks, T_f_, to fit the figure. Concentration (wt%), heat flow (mW), and direction of temperature change (3 K/min) are indicated. (B). Image of frozen ‘3-dimensional' 20 wt% CA at ~211 K. Si marks a silicon substrate. (C). Magnified thermograms from panel (A). The *T_tr2_*-transition is a net thermal effect produced by the resumed slow freezing of FCS_2_ and reverse glass-FCS_1_ transition, *T_g1,w_* (see text). Open arrow marks the temperature at which the resumed slow freezing ceases (see text). The meaning of other symbols is given in the text. (D, E). Images of frozen ‘2-dimensional' 10 wt% CA and 52 wt% CA at ~210 and 200 K, respectively. Bright spots are the parts of ice in contact with a cover glass. Arrows mark the channels of FCS_1_, ice, and a borderline of FCS_2_ (see text and movies S1, S2). (F). Image of frozen ‘2-dimensional' 55 wt% CA at ~221 K shows that freezing begins from multiple ice nucleating events (movie S3).

Figure 1A displays the thermograms of ‘3-dimensional' 10, 30, and 55 wt% CA solutions. Exothermic, *T_f_*, and endothermic, *T_m_*, peaks are produced by the enthalpy of fusion emitted during the freezing to pure ice and absorbed during equilibrium ice melting^46^, respectively. Regions without transitions are seen as a straight baseline. The different shape of *T_f_*-peaks shows that freezing is a *fast* process in diluted solutions and is hindered by increasing viscosity in a concentrated 55 wt% CA solution. The long low-temperature tail of *T_m_*-peaks indicates that ice starts to melt gradually from an ice/FCS interface where FCS concentration is largest. In OC-M observations of the freezing process of ‘3-dimensional' solutions, we always observe an abrupt dark flash produced by freezing. In Figure 1B, an OC-M image of a frozen ‘3-dimensional' solution shows that ice morphology and FCS are not distinguishable.

**Figure 2:**
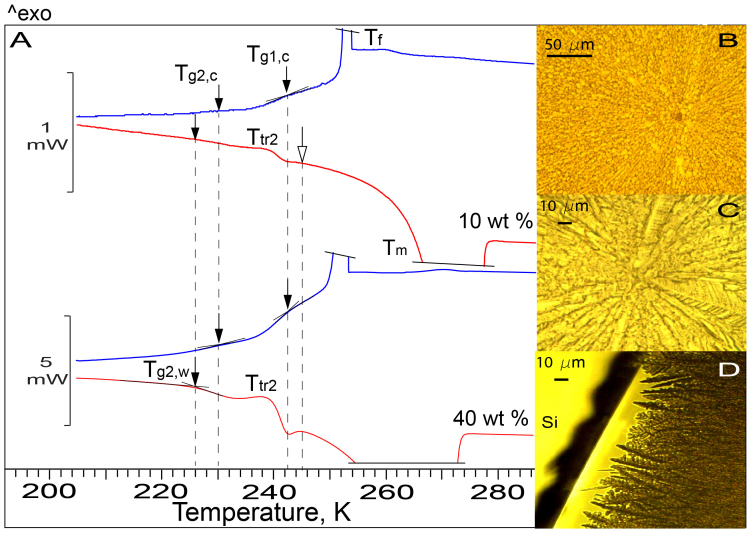
DSC thermograms and OC-M images of sucrose/H_2_O. (A). The thermograms are obtained from ‘3-dimensional' 10 and 40 wt% sucrose. All symbols have the same meaning as in Figure 1C. (B, C) Images of ‘2-dimensional' 10 wt% and 5 wt% sucrose taken at ~253 K demonstrate that freezing is triggered heterogeneously from a single ice nucleating event. Spherulitic IF is seen as bright tortuous ice needles/plates interweaved with the dark spots/channels of FCS_1_ (see also Figure 1D). The images of 10 wt% (B), 5 wt% (C), and 40 wt% sucrose (D) show how IF morphology changes with increasing concentration.

Magnified thermograms in Figure 1C for CA/H_2_O (and in Figure 2A for sucrose/H_2_O) are more informative than the thermograms in Figure 1A. In addition to the fast freezing process, *T_f_*-peak, which produces the majority of ice, the magnified cooling thermograms reveal a *slow* freezing process^13^, which manifests itself through an inclined exotherm on the cold side of *T_f_*-peak. We observed the fast and slow freezing processes also in OC-M measurements of ‘2-dimensional' solutions of all concentrations, including the solutions whose thermograms are presented in Figures 1 and 2 (movies S1 and S4). The cooling thermograms also reveal two liquid-glass transitions, *T_g1,c_* and *T_g2,c_*, which are recognized by the appearance of two steps, *ΔC_p,1c_* and *ΔC_p,2c_*, produced by the heat capacity change^46^. The *ΔC_p,1c_* and *ΔC_p,2c_* steps are only very subtle in the thermograms of 10 wt% CA (and 10 wt% sucrose in Figure 2A) because of the small amount of FCS formed. The warming thermograms reveal a reverse glass-liquid transition, *T_g1,w_*, and the *T_tr2_*-transition^9^,^10^,^11^,^12^,^29^,^30^,^31^,^38^,^39^. The existence of two liquid-glass transitions upon cooling of CA/H_2_O and sucrose/H_2_O and the *T_tr2_*-transition during the warming of frozen CA/H_2_O, to our best knowledge, has not been reported before.

## Discussion

The existence of *T_g1,c_* and *T_g2,c_* upon cooling requires the existence of two reverse glass-liquid transitions upon warming and, consequently, the formation of two FCS regions of different concentrations during freezing. The fact that *T_g1,c_*-transition is on the inclined thermogram (Figures 1C and 2A) suggests that the slow freezing and *T_g1,c_*-transition occur simultaneously, which also requires the existence of two FCS regions of different concentrations. In OC-M images of frozen ‘2-dimensional' CA/H_2_O and sucrose/H_2_O, the first region, FCS_1_, is seen as dark tortuous channels/pockets in between bright tortuous ice twigs (Figures 1D and 2B) or ice needles/plates (Figure 2C). OC-M images also demonstrate that supercooled diluted solutions freeze heterogeneously from a *single* ice nucleating event triggered by a foreign particle (Figure 2B) or substrate (Figure 2C). After nucleation, ice propagates rapidly as radial ~2–4 μm-thick tortuous twigs which form a *continuous* ice framework (IF) immersed into FCS_1_. We also observe that, as concentration increases, IF becomes a dendritic multi-branching pattern (Figures 1E and 2D). This dendritic morphology arises from growth instabilities brought about by insufficiently fast latent heat conduction and solute exclusion from ice during fast freezing. Concentrated solutions freeze from multiple ice nucleating events (Figure 1F and movie S3). Thus, our OC-M observations demonstrate that freezing supercooled solutions produce a continuous IF immersed into FCS_1_ and not isolated ice crystals, as has previously been believed.

The second, less concentrated (see below) region, FCS_2_, is formed in front of the advancing IF/FCS_1_ front and envelops the entire IF/FCS_1_ (movie S2, Figures 1D, 1E and 2D). Due to the limited rate of low-temperature diffusion of H_2_O to ice, a concentration gradient is established between FCS_1_ and FCS_2_. However, the volume of the transition region is much smaller than that of FCS_1_ and FCS_2_ and, consequently, only *T_g1,c_* and *T_g2,c_* are visible in the thermograms.

In OC-M measurements, we observe that, as temperature decreases, the slow freezing of FCS_2_ slows down due to increasing viscosity and ultimately ceases at ~208 K in CA/H_2_O and ~230 K in sucrose/H_2_O (movies S1 and S4). The fact that these temperatures coincide with the onset of liquid-glass transition, *T_g2,c_*, (Figures 1C and 2A), indicates that the FCS_2_ is associated with the *T_g2,c_*-transition and, consequently, it is less concentrated than FCS_1_, which itself vitrifies at *T_g1,c_*. Upon subsequent warming, the slow freezing resumes also at ~208 K and ~230 K in CA/H_2_O and sucrose/H_2_O, respectively (movies S1 and S4). In warming thermograms, these temperatures are the end of *reverse* glass-FCS_2_ transition, *T_g2,w_* (Figures 1C and 2A), where the viscosity of FCS_2_ has decreased sufficiently for resumed slow ice growth.

**Figure 3:**
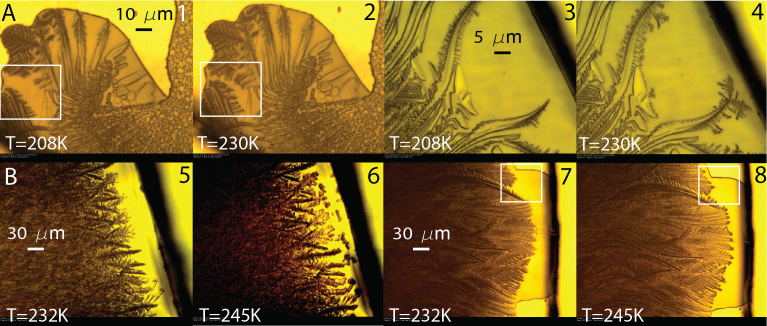
OC-M images of frozen CA/H_2_O and sucrose/H_2_O. (A). Pairs of images (1, 2) taken from ‘2-dimensional' 10 wt% CA and (3, 4) from 20 wt% CA show that upon warming of frozen solutions the resumed slow freezing of FCS_2_ continues to ~230 K. (B). Pairs of images (5, 6) taken from 40 wt% sucrose and (7, 8) from 45 wt% sucrose show that the resumed slow freezing continues to ~245 K. Squares show locations where the resumed ice growth is visible best.

In Figure 3, we present the images which captured the onset and end of the resumed slow freezing of FCS_2_ upon warming. They show that the resumed freezing continues to ~230 K in CA/H_2_O and ~245 K in sucrose/H_2_O (movies S1 and S4) i.e., it completely covers the temperature region of the *T_tr2_*-transition (Figures 1C and 2A). From this fact and from what was stated above, we conclude that the *T_tr2_*-transition is a net thermal effect produced by the resumed slow freezing of FCS_2_, which is responsible for the exothermic feature of the *T_tr2_*-transition, and reverse glass-FCS_1_ transition, *T_g1,w_*, which produces the *ΔC_p,1w_*-step. This solves the long-standing problem of the *T_tr2_*-transition and accounts for the appearance of “non-reversing” (crystallization) and “reversing” (glass transition) events in modulated DSC scans of the *T_tr2_*-transition^9^,^30^,^38^. We determine the onset temperature of *T_g1,w_*-transition at ~217 for CA/H_2_O and ~239 K for sucrose/H_2_O.

Both *T_g1,_*_w_ and *T_g2,_*_w_ are characteristic and reproducible temperatures which are independent of the initial solution concentration, as has been seen before for carbohydrate solutions^10^. Since *T_g_* increases with concentration and *T_g1,_*_w_ > *T_g2,_*_w_, we relate the *T_g1,_*_w_ to the glass transition of *maximally* FCS, *T_g_'*, and the concentration of FCS_1_ to the *maximal* freeze-concentration, *C_g_*'^38^. This solves another long-standing problem, namely, the problem of *T_g_'* and *C_g_*'. We calculate *C_g_*' ≈ 81 wt% and *C_g2,w_* ≈ 75 wt% for CA/H_2_O and *C_g_*' ≈ 85 wt% and *C_g2,w_* ≈ 81 wt% for sucrose/H_2_O using the Gordon-Taylor approach^47^,^48^. In the calculations, we use *T_g,CA_* = 284 K for pure CA^43^, *T_g,S_* = 335 K for pure sucrose^48^, and the Gordon-Taylor parameter of k_GT_ ≈ 5.43 for sucrose/H_2_O^48^ and our calculated k_GT_ ≈ 3.46 for CA/H_2_O. Our value of *C_g_*' ≈ 85 wt% for sucrose/H_2_O is larger than the literature data of *C_g_*' ≈ 82 wt%^38^.

**Figure 4:**
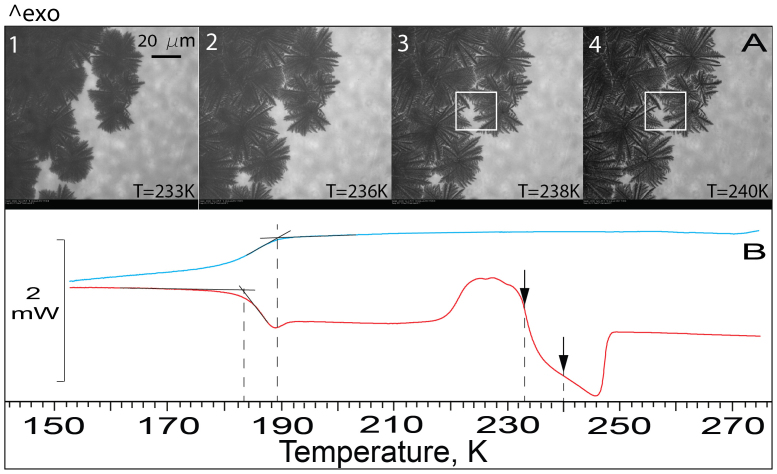
OC-M images and DSC thermograms of 62 wt% CA. (A). Images (1–4) are taken upon warming of ‘2-dimensional' 62 wt% CA previously cooled to 173 K. We observed no freezing upon cooling. Upon warming, freezing starts at ~220 K from multiple ice nucleating events (movie S5) and continues to ~240 K (image 4). Ice melting in FCS_1_, beginning at ~233 K (image 1), is seen as increasing brightness of IF/FSC_1_ region. Between ~233 and 240 K freezing and ice melting occur simultaneously. (B). The cooling thermogram of ‘3-dimensional' 62 wt% CA shows no indication of freezing, as in ‘2-dimensional' 62 wt% CA. A liquid-glass transition occurs at ~189 K. Upon warming, freezing starts also at ~220 K. Arrows show the temperature region in which freezing and melting occur simultaneously.

The fact that the resumed slow freezing continues to ~230 and 245 K (Figures 1C and 2A) implies that upon warming of frozen CA/H_2_O and sucrose/H_2_O, the fraction of FCS_2_ remains in liquid phase above *T_c_* ≈ *T_g_'* + 2 K. This suggests that if the freezing behaviour of pharmaceutical formulations is similar to that described above, then the remaining liquid FCS_2_ can form a ‘skin' on top of formulations and resist the vapour flow of sublimated ice during primary drying at *T_p_* < T_c_. Further, in the case of CA/H_2_O, the resumed freezing of FCS_2_ and ice melting at ice/FCS_1_ interface occur simultaneously between ~220 and 230 K, because this temperature range is well on the ice melting endotherm (Figure 1C). The resumed freezing increases FCS_2_ concentration, whereas ice melting at the ice/FCS_1_ interface, which starts at the end of the *T_g1,_*_w_-transition at ~220 K, decreases FCS_1_ concentration. Above ~230 K, when the two concentrations become equal, only ice melting continues. In sucrose/H_2_O, the annihilation of the concentration gradient between FCS_1_ and FCS_2_ takes place between ~242 and 245 K (Figure 2A). Simultaneous freezing and ice melting are best seen upon warming of ‘2-dimensional' 62 wt% CA previously cooled to 173 K (Figure 4 and movie S5).

A natural question may arise concerning the extent to which conclusions about the ice/FCS morphology of the bulk ‘3-dimensional' solutions can be drawn from the OC-M data of ~5–10 μm-thick solutions, which we call ‘2-dimensional' solutions only in order to distinguish them from large drops. The necessity to use micrometer-scaled thick solutions arises because one can only focus on approximately one micron-thick layer in the optical microscopy technique. We emphasize that our ‘2-dimensional' solutions are very different from the thin films of just a few molecular layers thickness. Whereas the physics and chemistry in such thin films is dominated by surface processes, our ‘2-dimensional' solutions are large (1 cm in diameter, see Methods section) and thick enough to behave as bulk solutions and, consequently, produce the ice/FCS morphology similar to that in bulk samples. Besides the parallels in the DSC and OC-M data discussed above, this is, e.g., also confirmed by the similarity of our pictures in Figures 1D and 2B and pictures obtained with a cryo-scanning electron microscope (C-SEM) in Figure 6 in Ref. 9. Our pictures show that below T_g_', FCS_1_ is amorphous (glassy) and represents a porous matrix (cake) with the pores filled with ice. Similarly, pictures in Figure 6 in Ref. 9 show an amorphous porous cake which was obtained after ice sublimation at 238 K < T_g_' ≈ 239 K from frozen ‘3-dimensional' 40 wt% sucrose. Thus our ‘2-dimensional' and ‘3-dimensional' solutions freeze similarly and produce a continuous IF (not isolated ice crystals as previously believed) immersed into FCS_1_ + FCS_2_.

In conclusion, this study introduces the ‘2-dimensional-solution' strategy as an approach for the visualization of freezing process *in situ* and, consequently, the determination of ice/FCS morphology of frozen biopharmaceutical formulations. Together with DSC measurements of ‘3-dimensional' bulk solutions this strategy solves the long-standing problems of the *T_tr2_*-transition of frozen hydrocarbon solutions and allows the practically precise determination of the critical formulation parameters *T_g_'*, *T_c_* and *T_p,_* the knowledge of which is crucial for the optimization of lyophilisation process^4^,^35^. Our findings suggest that a continuous IF may also be formed upon freezing of biological cells and organs that may give a new impetus to investigation of the resistance of living matter to freezing and its survival at low temperatures.

## Methods

We prepared 10–62 wt% citric acid, (C_6_*H*_8_O_7_), and 10–45 wt% sucrose (C_12_H_22_O_11_) solutions by dissolution of 99.5% CA (Merck) and 99.5% sucrose (Sigma) in ultrapure water. The freezing behaviour of approximately semi-spherical drops (5.5–6.5 mg), which in the text are referred to as ‘3-dimensional' solutions, were studied with a Mettler DSC822 calorimeter. The drops were cold-sealed in Al crucibles of 40 μl and studied at a cooling/warming rate of 3K/min between 320 and 163 K. Calorimeter calibration and details about DSC measurements are described elsewhere^13^,^14^,^23^. We also employed ~5–10 μm-thick films of CA/H_2_O and sucrose/H_2_O (referred to as ‘2-dimensional' solutions) for *in situ* observation of freezing/melting processes with an optical cryo-microscope (Olympus BX51) equipped with a Linkam cold stage and Linksys32 temperature control and video capture software. The solution films were formed between a *Si*-wafer and a cover-glass of 1 cm in diameter. Cryo-microscope measurements were performed at cooling/warming rate of 3 and 5 K/min between 320 and 163 K. The temperatures of heterogeneous freezing and melting of ‘3-dimensional' drops and ‘2-dimensional' solutions of the same concentration were quite similar, as expected, because ~5–10 μm-thick solutions are large/thick enough to behave as bulk solutions. More than 300 measurements performed with DSC and OC-M showed very good reproducibility of results.

## Author Contributions

A.B. designed the research, performed DSC and OC-M measurements and calculations, collected and analyzed data, and wrote the manuscript. N.B. performed some of DSC measurements, discussed results, and contributed to writing the manuscript. M.J.M., H.T., E.B. and T.L. discussed results and commented on the manuscript.

## Supplementary Material

Supplementary Information Legends for videos in SI

Supplementary Information Video 1

Supplementary Information Video 2

Supplementary Information Video 3

Supplementary Information Video 4

Supplementary Information Video 5
