# Supplementary material for: Visualization of Freezing Process in situ upon Cooling and Warming of Aqueous Solutions
Source: Sci Rep. 2014 Dec 10;4:7414. doi: 10.1038/srep07414 (PMC4261172; doi:10.1038/srep07414)
Supplement: Supplementary Information — Legends for videos in SI [file srep07414-s1.doc]

**Visualization of Freezing Process *in situ* upon Cooling and Warming of Aqueous Solutions**

Anatoli Bogdan^1,2,3*^, Mario J. Molina^4^, Heikki Tenhu^2^, Erminald Bertel^1^, Natalia Bogdan^5^ & Thomas Loerting^1^

^1^Institute of Physical Chemistry, University of Innsbruck, Innrain 52a, A-6020, Innsbruck, Austria.

^2^Laboratory of Polymer Chemistry, Department of Chemistry, University of Helsinki, P.O. Box 55, FIN-00014, Helsinki, Finland.

^3^Department of Physical Sciences, University of Helsinki, P.O. Box 64, FI-00014, Helsinki, Finland.

^4^Department of Chemistry and Biochemistry, University of California, San Diego, La Jolla, CA 92093-0356, USA.

^5^Faculty of Medicine, University of Helsinki, P.O. Box 63, FIN-00014, Helsinki, Finland.

## Legends for videos in SI

1. Video 1: The fast and slow freezing and subsequent melting of 20wt% CA (citric acid).

Video 1: Video 1 (10.8 MB) demonstrates the fast and slow freezing of 20wt% CA upon cooling. The slow freezing occurs in FCS_2_ and is terminated by the onset of the liquid-FCS_2_-glass transition at ~208K. Upon warming, the slow freezing recommences above the reverse glass-liquid-FCS_2_ transition at ~208K and proceeds to ~230K. Above ~230K ice only melts. Temperature change is seen in the left bottom corner.

1. Video 2: The formation of FCS_2_ in front of the advancing IF/FCS_1_ front in freezing 40wt% CA.

Video 2: Video 2 (4.22 MB) demonstrates the formation of FCS_2_ in front of the advancing IF/FCS_1_ front during the freezing of 40wt% CA. In the left upper corner, the FCS_2_/air borderline moves when the IF/FCS_1_ front propagates.

1. Video 3: The freezing of 58wt% CA from multiple ice nucleating events.

Video 3: Video 3 (3.99 MB) demonstrates that 55wt% CA freezes from multiple ice nucleating events. Dark dots are ice crystals formed by vapour deposition on the outer side of a cover glass.

1. Video 4: The fast and slow freezing and subsequent melting of 40wt% sucrose.

Video 4: Video 4 (15.8 MB) demonstrates the fast ta slow freezing of 40wt% sucrose upon cooling. The slow freezing occurs in FCS_2_ and is terminated by the onset of the liquid-FCS_2_-glass transition at ~230K. Upon warming, the slow freezing recommences above the reverse glass-liquid-FCS_2_ transition at ~230K and proceeds to ~245K. Above ~245K ice only melts.

1. Video 5: Simultaneous freezing and ice melting upon warming of 62wt% CA.

Video 5: Video 5 (6.95 MB) demonstrates simultaneous slow freezing in FCS_2_ and ice melting in FCS_1_ upon warming of 62wt% CA previously cooled to 173K. The freezing starts at ~220K and proceeds to ~240K. Ice melting in FCS_1_ starts at ~233K and is seen as the increasing brightness of IF/FCS_1_. Between ~233K and ~240K the freezing in FCS_2_ and melting in FCS_1_ proceed simultaneously.
